# Rapid and Unambiguous Detection of DNase I Hypersensitive Site in Rare Population of Cells

**DOI:** 10.1371/journal.pone.0085740

**Published:** 2014-01-21

**Authors:** Wei-ping Zeng, Margaret M. McFarland

**Affiliations:** 1 Department of Biochemistry and Microbiology, Marshall University, Huntington, West Virginia, United States of America; 2 Department of Paediatrics, Joan C. Edwards School of Medicine, Marshall University, Huntington, West Virginia, United States of America; 3 Centre for Cell Development and Differentiation, Department of Biology, College of Science, Marshall University, Huntington, West Virginia, United States of America; University of Bonn, Institut of experimental hematology and transfusion medicine, Germany

## Abstract

DNase I hypersensitive (DHS) sites are important for understanding *cis* regulation of gene expression. However, existing methods for detecting DHS sites in small numbers of cells can lead to ambiguous results. Here we describe a simple new method, in which DNA fragments with ends generated by DNase I digestion are isolated and used as templates for two PCR reactions. In the first PCR, primers are derived from sequences up- and down-stream of the DHS site. If the DHS site exists in the cells, the first PCR will not produce PCR products due to the cuts of the templates by DNase I between the primer sequences. In the second PCR, one primer is derived from sequence outside the DHS site and the other from the adaptor. This will produce a smear of PCR products of different sizes due to cuts by DNase I at different positions at the DHS site. With this design, we detected a DHS site at the *CD4* gene in two CD4 T cell populations using as few as 2×10^4^ cells. We further validated this method by detecting a DHS site of the *IL-*4 gene that is specifically present in type 2 but not type 1 T helper cells. Overall, this method overcomes the interference by genomic DNA not cut by DNase I at the DHS site, thereby offering unambiguous detection of DHS sites in the cells.

## Introduction

Chromatin remodeling is a characteristic of cell lineage specification and cellular response to external stimulation. During these processes, the cells adopt an “open” structure at certain chromatin regions. These chromatin regions can be detected by virtue of their hypersensitivity to DNase I digestion, and hence are known as DNase I-hypersensitive (DHS) sites [1]. The DHS sites often contain *cis* regulatory elements associated with gene activation such as locus control regions, enhancers and boundary elements, but could also contain silencers [2], [3]. Therefore, identifying DHS sites and comparing their differences in different cell types or the same cells under different experimental treatments is key to understanding gene regulation under the different circumstances.

In a mammalian cell, up to 3% of the genome may be DNase I hypersensitive [4]. In recent years, microarray and deep sequencing methods have been successfully used to map genome-wide distribution of the DHS sites in a given cell type [5], [6]. The genome-wide map of the DHS sites provides a valuable starting point of research focusing on a specific biological question impacted by the regulation of a given gene or a set of genes. For example, one may need to know the changes in DHS sites at a particular gene locus identified by the genome-wide approaches in response to different experimental treatments. Alternatively one may wish to determine whether certain DHS sites identified in one cell type by the genome-wide approaches are also present in other cell types. For such studies, whole-genome analysis may be cost inhibitory and unnecessary. The traditional Southern-blot based method of DHS analysis is not only cumbersome but also impractical when the cell numbers are limited. Here a simple reliable method is described for unambiguous determination of DHS sites in rare populations of cells.

## Materials and Methods

### Animals and Cells

All animal work is approved by the Marshall University IACUC. Genetically modified mouse strain C.Cg-Foxp3tm2Tch/J [7] was purchased from Jackson Laboratory. Total CD4 T cells were prepared by negative selection as previously described [8]. The total CD4 T cells were stained with fluorescence labelled anti-CD4 (APC), anti-CD62L (PE). Naïve CD4 conventional T (Tcon) cells (CD4^+^CD62L^high^GFP^−^) and natural regulatory T (nTreg) (CD4^+^CD62L^high^GFP^+^) cells were isolated by fluorescence activated cell sorting (FACS). For the differentiation of type 1 and type 2 T helper (Th1 and Th2) cells, sorted naïve CD4 Tcon cells were stimulated with T cell-depleted spleen cells plus anti-CD3 antibodies under Th1- and Th2-polarizing conditions as previously described [9].

Primary mouse fibroblasts were derived from skin similarly to described before [10], [11]. Briefly, shaved skin was cut into pieces of 1 mm^2^ size, and digested with 200 mg/ml collagenase (Sigma Aldrich) in HBSS at 37°C for 20 min with rotation. The digested skin fragments were collected by centrifugation, washed once with Hank’s balanced salt solution (HBSS) then seeded in tissue culture dishes in Eagle’s minimum essential medium (EMEM) plus 10% fetal bovine serum (FBS) for 7–10 days. Fibroblasts exited from the skin fragments were harvest by trypsin treatment followed by trypsin inactivation with 10% FBS. The cells were washed 3 times with 1× phosphate balanced saline (PBS) before they were used for nuclei isolation.

### Nuclei Preparation and DNase I Digestion

Nuclei preparation and DNase I digestion were carried out as previously described with modifications [12]. Up to 2×10^5^ cells were lysed on ice in 2 ml ice-cold lysis buffer (10 mM Tris pH 7.4, 15 mM NaCl, 5 mM MgCl_2_, 10 mM EDTA, 60 mM KCl, 0.2% NP-40, 0.5 mM DTT, 0.5 µM Spermidine, 300 mM sucrose) supplemented with 1× protease inhibitors (Roche). After 5 min on ice, 8 ml of lysis buffer containing 0.1 mM EDTA, no sucrose and protease inhibitors, was added to the tube, and the nuclei were centrifuged at 500 *g* for 10 min at 4°C with deceleration off. The nuclei were washed once in lysis buffer containing 0.1 mM EDTA, no sucrose and protease inhibitors, once in 1× DNase I buffer (15 mM NaCl, 5 mM MgCl_2_, 60 mM KCl, 0.1 mM EGTA, 10 mM Tris-HCl, pH 7.4). The nuclei were then resuspended in 40 µl 1× DNase I buffer supplemented with 300 mM sucrose and transferred to a thin wall 200 PCR tube. Various amounts of DNase I (Worthington) diluted in 10 µl 1× DNase I buffer were added to the nuclei, and mixed 3 times with a large-orifice tip. The mixture was incubated on ice for 1 hr. At the end of the incubation, 50 µl of 1% low melt agarose (dissolved in 100 mM EDTA) was added and mixed with a large-orifice tip.

### In-gel Purification and Blunt-ending of Genomic DNA

In-gel genomic DNA isolation and blunt-ending were performed as previously described [13]. Briefly, the gel plug was released from the PCR tube by cutting open the sidewall with a scalpel. The gel plug was incubated with LIDS buffer (10 mM Tris-HCl, pH 8.0, 100 mM EDTA, 1% lauryl sulphate lithium salt) as described. Afterwards, the plug was washed with 50 mM EDTA 5 times and 1× NEB2 (Biolabs) once. The plug was then transferred to a mixture of 78.6 µl H_2_O, 10.4 µl 10× NEB2, 5 µl 10 mM dNTPs, 1× bovine serum albumin (BSA) (Biolabs), 0.1 M DTT and 6 µl T4 DNA polymerase (Biolabs), and incubated at room temperature for 4 hr.

### Adaptor Ligation

After blunt-ending, T4 DNA polymerase solution was removed from the gel plug. The gel plug was incubated with 0.75 ml TE buffer (1 mM Tris-HCl, pH 8.0, 1 mM EDTA) on ice for 10 min, then 50 µl 1× ß-agarase buffer at 75°C for 20 min to overnight. After the temperature cooled down to 42.5°C, 1 unit ß-agarase (Biolabs) was added to the liquidated gel plug, mixed and incubated for 2 hr. The digested gel plug solution was phenol-chloroform extracted, and DNA was ethanol precipitated together with 20 µg glycogen carrier. For adaptor ligation, the DNA was dissolved in 36 µl H_2_O, mixed with 5 µl 1× T4 DNA ligase buffer (Fermentas), 6 µl 25 µM annealed biotinylated adaptor (see Table 1 for adaptor sequences) and 3 µl T4 DNA ligase, and incubated at 16°C overnight. After the ligation, the genomic DNA was separated from free adaptors by electrophoresis in 0.7% low melt agarose gel.

**Table 1:** Oligonucleotide Sequences.

Name	Sequence	Application
Adaptor 1	5′-Bio-ACAGGTTCAGAGTTCTACAGTCCGAC	Adaptor
Adaptor 2	5′-pGTCGGACTGTAG AACTCTGAAC-Amm-3′	Adaptor
P1	GGCTTCATGGCTCAGAACCTAC	1^st^ and 2^nd^ PCR for CD4 DHS
P2	CCCTCAGCTACTTTCTGTGACT	1^st^ PCR
P3	GTTCAGAGTTCTACAGTCCGAC	2^nd^ and 3^rd^ PCR for CD4 DHS; 2^nd^ PCR for IL-4 HS II
P4	TGCTTCCCACCTGCCCCCAAGCAT	3^rd^ PCR for CD4 DHS
IL4-1	GCCTTGCTTGATACGGTATCT	1^st^ and 2^nd^ PCR for IL-4 HS II
IL4-2	GCCTGTAGGGACCATACGA	1^st^ PCR for IL-4 HS II

Gel segment containing the high-molecular-weight DNA (>23 kb) was digested with ß-agarase and DNA was recovered by ethanol precipitation as described above.

### Magnetic Isolation of Adaptor Ligated DNA

The adaptor ligated DNA was sonicated to an average size of 500 bp with a Covaris sonicator following the manufacturer’s instructions. In the meantime, a 20 µl aliquot of avidin-conjugated magnetic beads Av-Dynal beads (Invitrogen) was washed twice with 1× TE buffer, once with 1× B&W buffer (5 mM Tris.HCl, pH 7.5, 0.5 mM EDTA, 1 M NaCl), then blocked with 1 µg/µl yeast tRNA and 0.1 µg/µl BSA in 30 µl 1× B&W buffer by rotating at 30°C for 1 hr. The beads were combined with the sonicated DNA in a total volume of 80 µl 1× B&W buffer supplemented with 1 µg/µl yeast tRNA and 0.1 µg/µl BSA. The mixture was rotated at 30°C for 30 min. The beads were separated from the supernatant on a magnetic stand. DNA from the supernatants were dialysed against 1× TE overnight and saved for later use. The beads were washed 5 times with 1× TE. After washes, the beads were treated twice with 200 µl 0.15 M NaOH at room temperature for 5 min. The beads were again washed 5 times with 1× TE, and suspended in 20 µl TE. DNA bound to the beads constituted the DHS library. DNA from the supernatants and the biotin end-labelled DNA fragments bound to the avidin-conjugated magnetic beads (the DHS libraries) will be used as templates in PCR.

### PCR Amplification

DHS library (5 µl of beads), DNA from the supernatants described above or total genomic DNA (300 ng) undigested by DNase I was used as templates for PCR. The primer sequences for all PCR reactions are listed in Table 1. PCR conditions were 95°C 3 min, (95°C 30 sec, 61°C 30 sec, 72°C 30 sec)×30 cycles, 72°C 5 min. SYBR green-based real-time PCR was performed using the same conditions for 40 cycles. Amplification signals relative to the sample indicated each experiment were calculated using the 2^−Δ*Ct*^ method [14]. Statistical significance of differences in amplification signals was determined by Student’s *t* test.

## Results

### Design of the Method

PCR based methods for detecting DHS sites are often the method of choice for experiments with multiple samples but limited materials. Currently available PCR-based DHS detection methods [12], [15] rely on quantitative differences of the PCR amplification of the DHS sequences between DNase I digested and undigested samples. However, for detecting DHS sites the nuclei should only be partially digested, so even in the DNase I-digested sample the majority of genomic DNA at a DHS site is in fact not cut by DNase I. Such a large proportion of uncut DNA can obscure the quantitative difference and lead to ambiguous results. To overcome this problem, we designed a method in which the DNase I-digested DNA are separated from undigested DNA and used as templates in 2 sets of PCR to detect DHS site.

**Figure 1:**
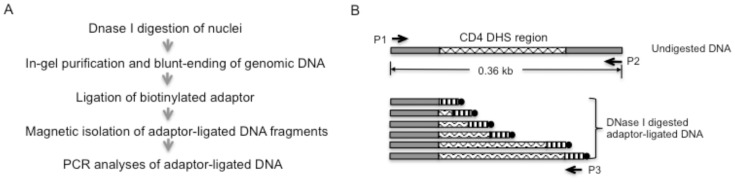
Method design. A. Summary of the major steps of the experimental procedure. B. Design of PCR detection of the CD4 DHS site. Short horizontal bars with vertical stripes represent the adaptor sequence. The dark spheres at the end of the adaptors represent biotin. Primer locations are indicated by the horizontal arrows.

We used the DHS region upstream of the mouse *CD4* gene as the model system to develop the new method. The upstream CD4 DHS region is well characterized in CD4 T cells, which consists of a DNA segment of about 280 bp [16]. Figure 1A summarizes the major steps of the new method. Nuclei are subjected to DNase I digestion then embedded in low-melt agarose gel. Genomic DNA are purified and blunt ended in gel. The genomic DNA derived from the DNase I-digested nuclei contain DNA fragments with ends generated by DNase I cuts randomly distributed at different positions within the DHS region. After the blunt-ending, a biotinylated adaptor is ligated to the DNase I-generated ends. After the ligation, high-molecular-weight (>23 kb) DNA is separated from free adaptor by electrophoresis. The DNA is recovered and sonicated to an average size of 500 bp. DNA fragments with DNase I-generated ends are isolated via the binding of biotin to avidin-conjugated magnetic beads. The isolated DNA is the DHS library of the CD4 T cells.

The DHS libraries are used as templates in two different PCR reactions (Fig. 1B). In the first PCR, forward and backward primers are derived from sequences up- and downstream of the CD4 DHS site, respectively. In addition to the DHS libraries, total genomic DNA undigested with DNase I is used as control templates in the PCR. The control DNA templates are expected to give rise to a 0.36 kb PCR product. In contrast, the DHS library templates are expected not to produce any PCR products due to the cuts of the templates by DNase I between the forward and backward PCR primers. Thus a negative PCR result represents the presence of the DHS site. However, a negative PCR result can be caused by a variety of reasons, for example the lack of DNA bound to the beads. Therefore, the second PCR is designed to confirm the presence of the DHS site by a positive PCR result. The second PCR is performed with the same forward primer, however the backward PCR primer is derived from the adaptor sequence ligated to the DNase I-generated DNA ends. If the DHS is present, the second PCR products will be a smear of DNA of different sizes resulted from cuts by DNase I at different positions in the DHS region. Conversely, the control total genomic DNA templates would produce no PCR products.

### DNase I Digestion

**Figure 2:**
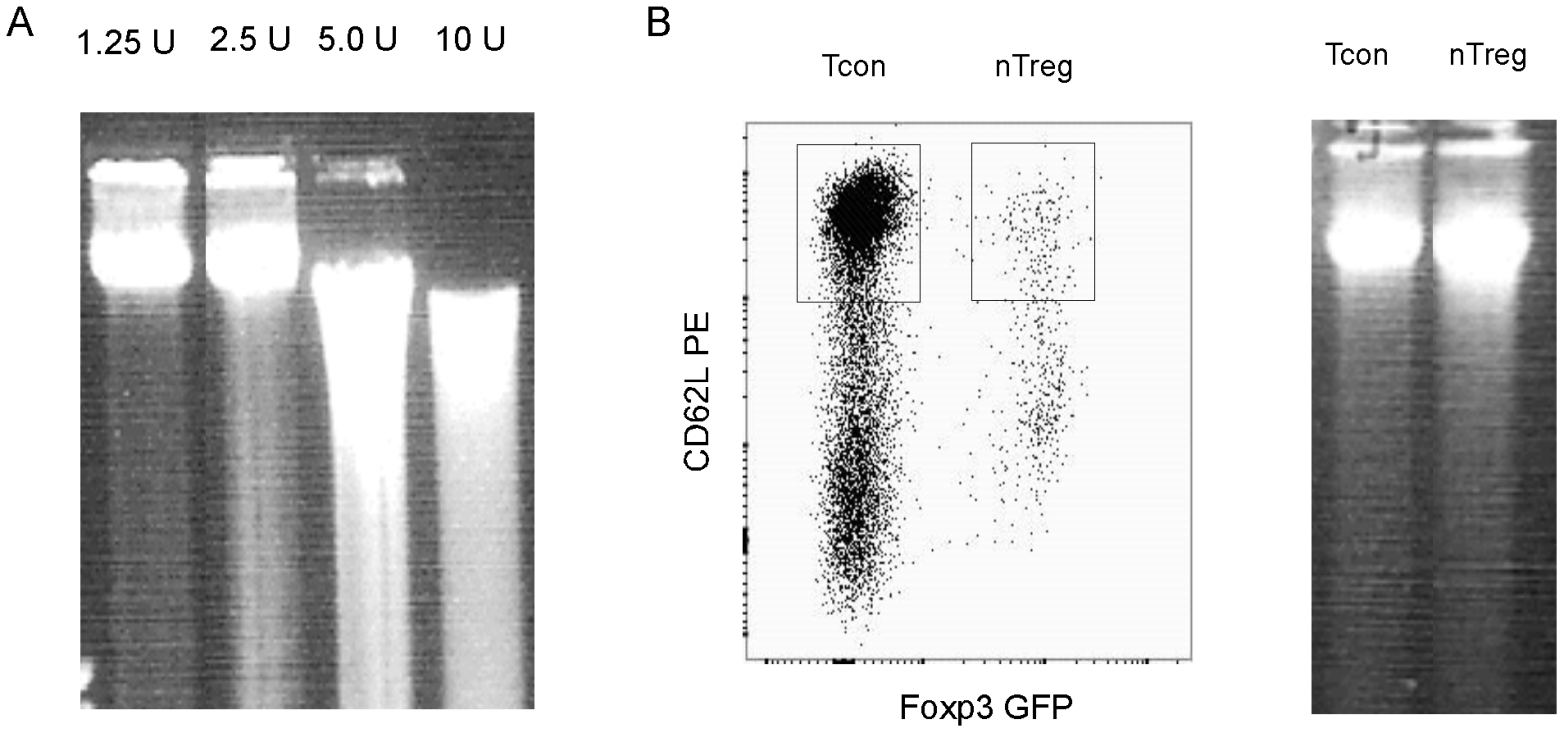
DNase I treatment of nuclei. A. Nuclei isolated from 2×10^5^ total CD4 T cells were treated with the indicated amounts of DNase I. After the treatment, the nuclei were embedded in low-melt agarose gel. Genomic DNA was in-gel purified then released from the gel plug and electrophoresed on a 0.7% agarose gel. B. Left panel shows the isolation of naïve CD4 Tcon cells and nTreg cells by FACS. In the right panel, the nuclei from the isolated cells were treated with 1.25 units of DNase I. In-gel purified genomic DNA was analysed as in A.

To work with rare populations of cells, it is necessary to pre-determine the conditions for DNAse I digestion using a relatively more abundant cell population. We therefore first used total CD4 T cells to pre-determine the amount of DNase I to be used in the experiments (Fig. 2A). To increase the reproducibility and minimize experimental variations, we chose to digest the nuclei of CD4 T cells with various amounts of DNase I on ice for a relatively long period of time (1 hr). After the digestion, nuclei were embedded in low-melt agarose gel. Genomic DNA was in-gel purified by stripping off proteins and other biomaterials. The DNA was then released from the gel plug by ß-agarase digestion, and analysed by gel electrophoresis (Fig. 2A). While genomic DNA from control nuclei not digested with DNase I could not enter the gel due to the large sizes (not shown), the majority of the genomic DNA derived from the DNase I digested nuclei could enter the gel. The degrees of digestion were directly proportional to the amounts of DNase I (Fig. 2A). In subsequent experiments, we chose to use 1.25 units of DNase I to digest nuclei unless indicated otherwise.

Naïve CD4 conventional T (Tcon) cells and natural regulatory T cells (nTreg) cells were used to develop the method of rapid detection of DHS sites. NTreg cells are a rare population of CD4 T cells that comprises only 5–10% of total CD4 T cells or 1–2% of total leukocytes in spleen and lymph nodes [17]. Both the CD4 Tcon and nTreg cells were isolated by FACS from C.Cg-Foxp3tm2Tch/J mice (Fig. 2B, left), in which the nTreg cells are marked by the expression of GFP [7]. The FACS isolated cells were immediately used to prepare nuclei. The nuclei were digested with 1.25 units of DNase I. After in-gel purification, the genomic DNA was released from the gel plug and analysed by electrophoresis (Fig. 2B, right). The degrees of digestion of both cell types were very similar to that of total CD4 T cells in Figure 2A, demonstrating the consistency of the outcome of the DNase I digestion.

### Preparation of DHS Libraries

**Figure 3:**
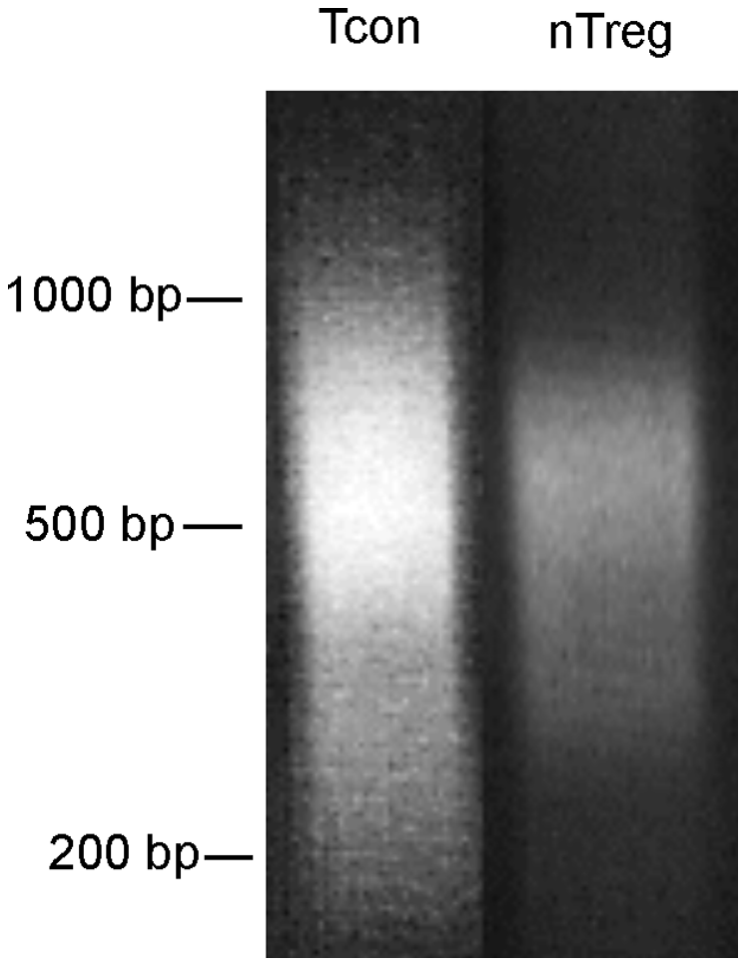
Sonication of adaptor ligated genomic DNA. After in-gel purification and blunt-ending, the genomic DNA of naïve CD4 Tcon and nTreg cells were ligated to biotinylated adaptor. The ligated DNA of high molecular weight (>23 kb) was gel purified to remove free adaptors, then fragmented to an average size of 500 bp by sonication. Gel picture of the sonicated DNA is shown.

After DNase I digestion, the genomic DNA was in-gel purified and blunt-ended. The DNA was released from the gel plug, purified and ligated to a biotinylated adaptor so that the DNA ends generated by DNase I digestion were attached to the adaptor and became biotinylated. The adaptor-ligated DNA was sonicated to an average size of 500 bp (Fig. 3). The sonicated DNA fragments were subject to isolation by avidin-conjugated magnetic beads in the presence of yeast tRNA and BSA to block nonspecific binding of the genomic DNA to the beads. After removing the supernatants, the beads were washed with TE buffer and treated with NaOH to remove residual nonspecific DNA bound to the beads. Adaptor ligated DNA fragments remaining bound to the beads were the DHS libraries.

### Detection of DHS Site Upstream of the *CD4* Gene

**Figure 4:**
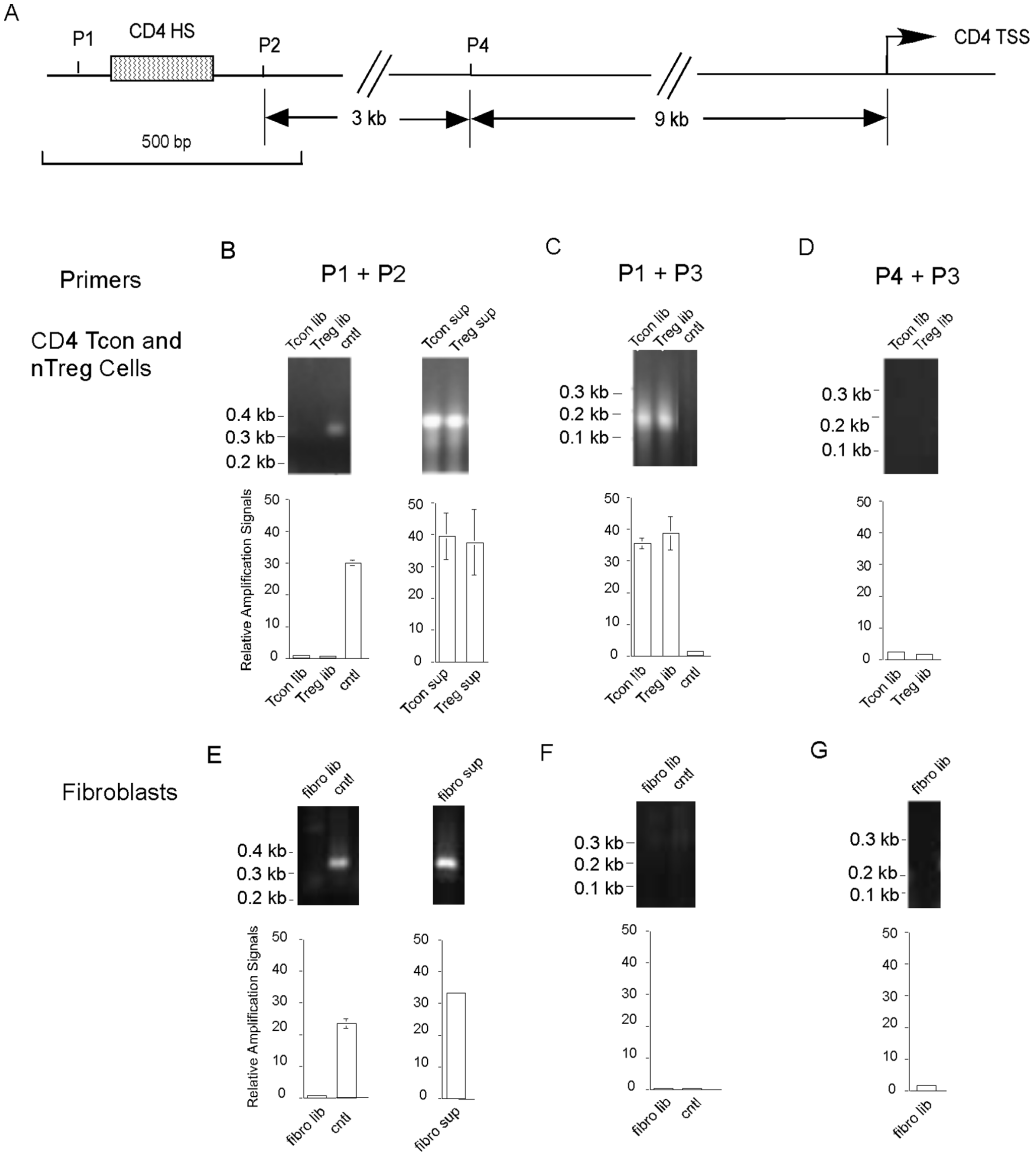
Detection of CD4 DHS site by PCR. DHS libraries or DNA in the supernatants after magnetic beads separation of naïve CD4 Tcon, nTreg cells (B–D) or primary fibroblasts (E–G) were used as templates for regular (upper panels) or real-time (lower panels) PCR analyses. Relative amplification signals in the real-time PCR were determined by comparison to the signals of DNase I-untreated total genomic DNA template amplified with primers P1 and P3 (P3 is complementary to the ligated adaptor). A. Schematic presentation of the positions of the CD4 DHS site, PCR primers and the transcription start site (TSS) of the *CD4* gene. B, E. First set of PCR using P1 and P2 primer pair. The left panel shows the PCR result using the beads-bound DHS libraries as templates; the right panel shows the PCR results using DNA remaining in the supernatants after beads isolation as templates. C, F. Second set of PCR using P1 and P3 primer pair. D, G. Third set of PCR using primer pair of P4 and P3. Tcon lib, naïve CD4 Tcon DHS library; Treg lib, naïve nTreg DHS library; Tcon sup: naïve CD4 Tcon supernatant; Treg sup, naïve nTreg supernatant; cntl, control total genomic DNA from total CD4 T cells not treated with DNase I; fibro lib, primary fibroblast DHS library; fibro sup: primary fibroblast supernatant.

We used both regular and real-time PCR to detect the CD4 DHS site in the naïve CD4 Tcon cells and nTreg cells following the experimental design outlined in Figure 1. The positions of the PCR primers at the *CD4* gene locus are shown in Figure 4A. The first PCR was performed using primer pair P1 and P2 derived from sequences up- and downstream of the CD4 DHS site, respectively. PCR using templates of the control undigested total genomic DNA produced an expected 360 bp product. In contrast, PCR using the templates of the DHS libraries (beads-isolated DNA) of both the naïve CD4 Tcon and nTreg cells did not produce such PCR product (Fig. 4B, upper panel, left). Real-time PCR results showed the quantitative differences of the PCR amplification signals using the different templates (Fig. 4B, lower panel, left), which were consistent with those of the regular PCR. When DNA in supernatants after the beads separation was used as templates, the first PCR detected the 360 bp band as in the control (Fig. 4B, upper panel, right). Again real-time PCR confirmed this result (Fig. 4B, lower panel, right). In this particular case, there were no statistically significant differences in the amplification signals between the supernatants and the control of total genomic DNA. The presence of the PCR product demonstrated that the nuclei of the naive CD4 Tcon cells and nTreg cells were not over digested by DNase I as plenty of DNA templates without a cut at the DHS site was still present in the supernatants.

In the second PCR, the forward primer was the same as that of the first PCR (P1), but the backward primer (P3) was derived from the adaptor sequence. As shown in Figure 4C upper panel, the PCR products of either cell type appeared as a smear on the agarose gel. The DNA smears were PCR products of different DNA sizes resulting from cuts by DNase I at different positions in the CD4 DHS site. As expected, the control templates did not generate any PCR product. Real-time PCR showed the same pattern of amplification (Fig. 4C lower panel). To further demonstrate the specificity of the detection of the CD4 DHS, we performed similar PCR using the same adaptor-derived backward primer (P3) but a different forward primer (P4) that was derived from a non-DHS region of the *CD4* locus (Fig. 4A). Both regular and real-time PCR showed no amplification (Fig. 4D). Thus, the PCR experiments specifically detected the CD4 DHS site in both niave CD4 Tcon cells and nTreg cells.

To demonstrate that the CD4 DHS site was detected only in the right cell types, we performed similar PCR experiments using mouse primary fibroblasts that do not express CD4. When the fibroblast DHS library was used as template, regular and real-time PCR using P1 and P2 primers showed no amplification (Fig. 4E, left). This was due to the fact that DNA at the *CD4* locus was not pulled down by the magnetic beads. In contrast, PCR using templates of DNA in the supernatant after beads purification produced the 360 bp product (Fig. 4E, right). Unlike in the CD4 T cells, second PCR using P1 and P3 primers produced no PCR products (Fig. 4F), neither did PCR using the P3 and P4 primer pair (Fig. 4G). Therefore, as expected the CD4 DHS site was not detected in mouse primary fibroblasts.

### Sequencing of the DNA Smears

**Figure 5:**
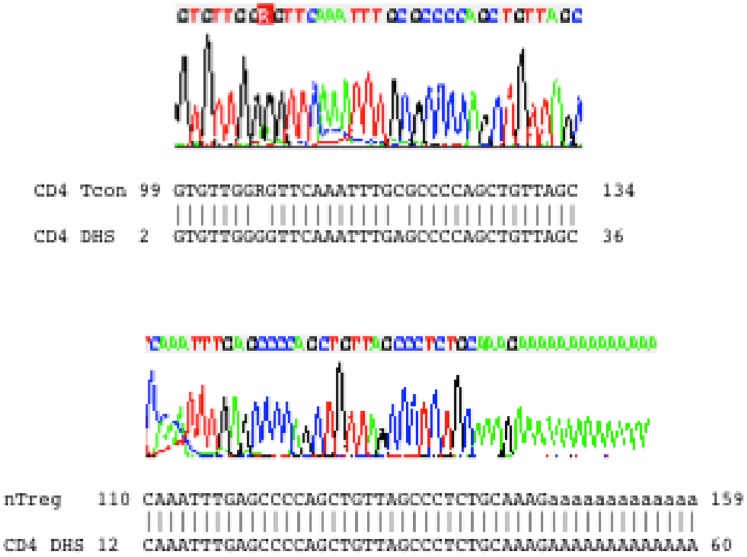
Sequencing verification of the smear DNA. DNA was purified from the smears in lane 1 and 2 in Figure 4C, and cloned in a pBluescript plasmid. Recombinant clones were sequenced, and the sequences were aligned to the CD4 DHS sequence. A representative clone for each population of the naïve CD4 Tcon and nTreg cells is shown.

To further confirm that the DNA smears observed in Figure 4C were indeed derived from the CD4 DHS sequence, the DNA smears were purified from the gel and cloned to a plasmid vector. Sequencing of randomly picked recombinant clones of these two cell populations showed that all contained inserts derived from the sequence of the CD4 DHS site. Partial sequences of a clone of either cell type and their alignments to the CD4 DHS sequence are shown in Figure 5.

### Determining the Minimum Cell Number

**Figure 6:**
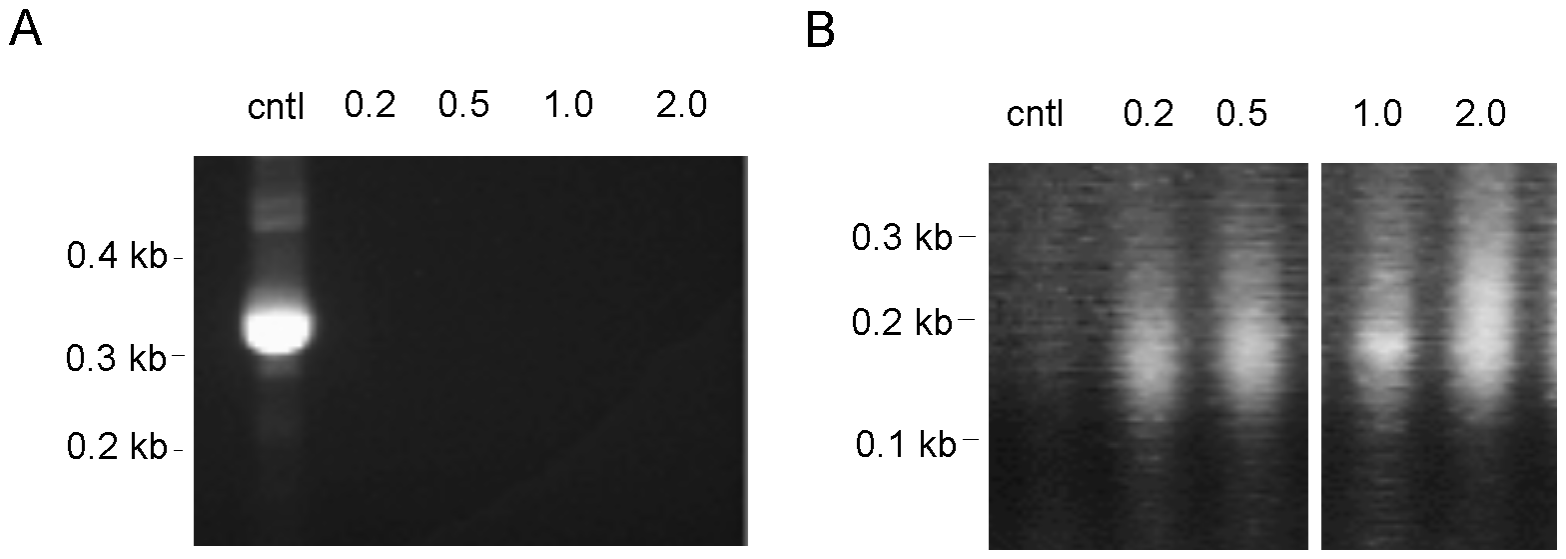
Determining the number of cells required for detecting a DHS site. Different numbers (x10^5^) of total CD4 T cells as indicated for each lane were used to prepare nuclei for DHS analysis. Magnetic beads-isolated DNA and control total genomic DNA (cntl) were used for the first (A) and second (B) PCR as in Figure 4.

In the above experiments 2×10^5^ cells were used for detecting the CD4 DHS site. We lowered the number of cells to determine the sensitivity of the method. Figure 6 shows the results of the first (left panel) and second (right panel) PCR using starting numbers of cells ranging from 0.2 to 2×10^5^. DNA smear of the second PCR products was clearly detected when as low as 2×10^4^ cells were used. We were not able to reliably detect the DHS site with fewer cells (data not shown).

### Validation of the Method

**Figure 7:**
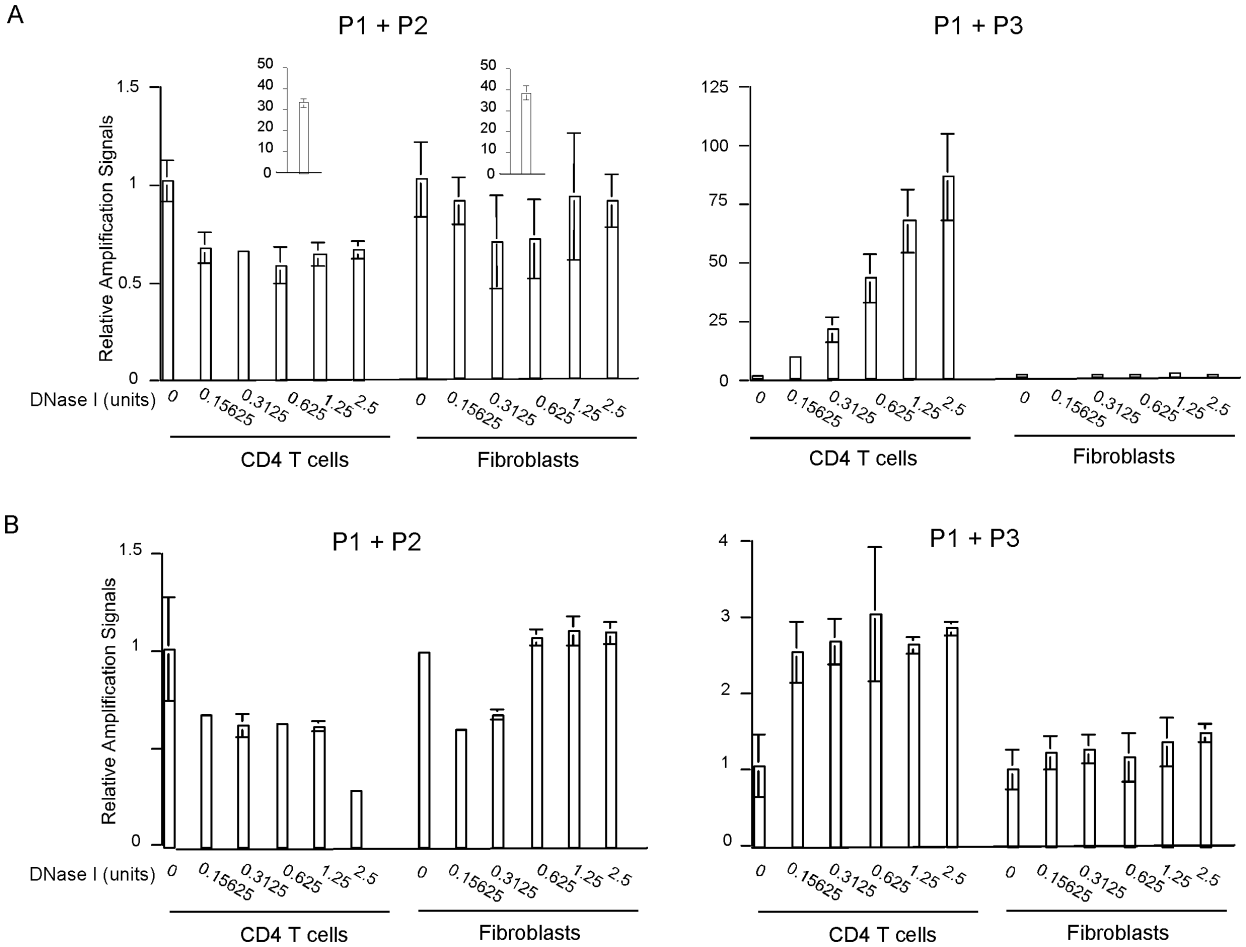
Detection of CD4 DHS site in total CD4 T cells and primary fibroblasts with different degrees of DNase I digestion. The CD4 DHS site was detected by real-time PCR with the indicated primer pairs. A. DHS libraries derived from nuclei of total CD4 T cells or primary fibroblasts digested with different amounts of DNase I were used as PCR templates. B. DNA in the supernatants after library isolation with magnetic beads were used as PCR templates. In all panels of the figure, relative amplification signals were determined by comparing to that of DNase I-undigested samples of the respective cell type. Insets show the amplification signals using DNase I-untreated total genomic DNA of the indicated cell types as templates.

First, we determined whether the CD4 DHS site could be detected using DHS libraries derived from nuclei digested with different amounts of DNase I. DHS libraries were prepared from total CD4 T cells and primary fibroblasts and used as templates in real-time PCR analyses. In the first PCR using primer pair P1 and P2, little amplification was observed in both cell types regardless of whether the nuclei were treated or untreated with DNase I (Fig. 7A, left). In contrast, in the second PCR using primer pair P1 and P3, strong amplification was detected in the DNase I treated samples of total CD4 T cells but not fibroblasts. The amplification signals appeared proportional to the amounts of DNase I (Fig. 7A, right).

We also used DNA in the supernatants after beads isolation of the DHS libraries as templates to perform the same experiments (Fig. 7B). When P1 and P2 were used as primers (Fig. 7B, left panel), modest but consistent decreases of PCR amplification signals were detected in all the DNase I-digested samples compared with the undigested sample of the CD4 T cells. In the fibroblast samples, there appeared to be some nonspecific reduction of amplification signals in two DNase I-digested samples but not the others. When P1 and P3 were used as primers (Fig. 7B, right panel), modest increases (2–3 fold) of amplification signals were detected in the DNase I-digested over undigested samples of the CD4 T cells. The increases likely resulted from residual adaptor-ligated DNA left in the supernatants. In contrast, no increase of PCR amplification was observed in DNase I-digested fibroblast samples. This outcome is consistent with the notion that the use of an exogenous primer (P3) is advantageous in detecting DHS site compared with PCR using both primers derived from endogenous genomic DNA sequences.

**Figure 8:**
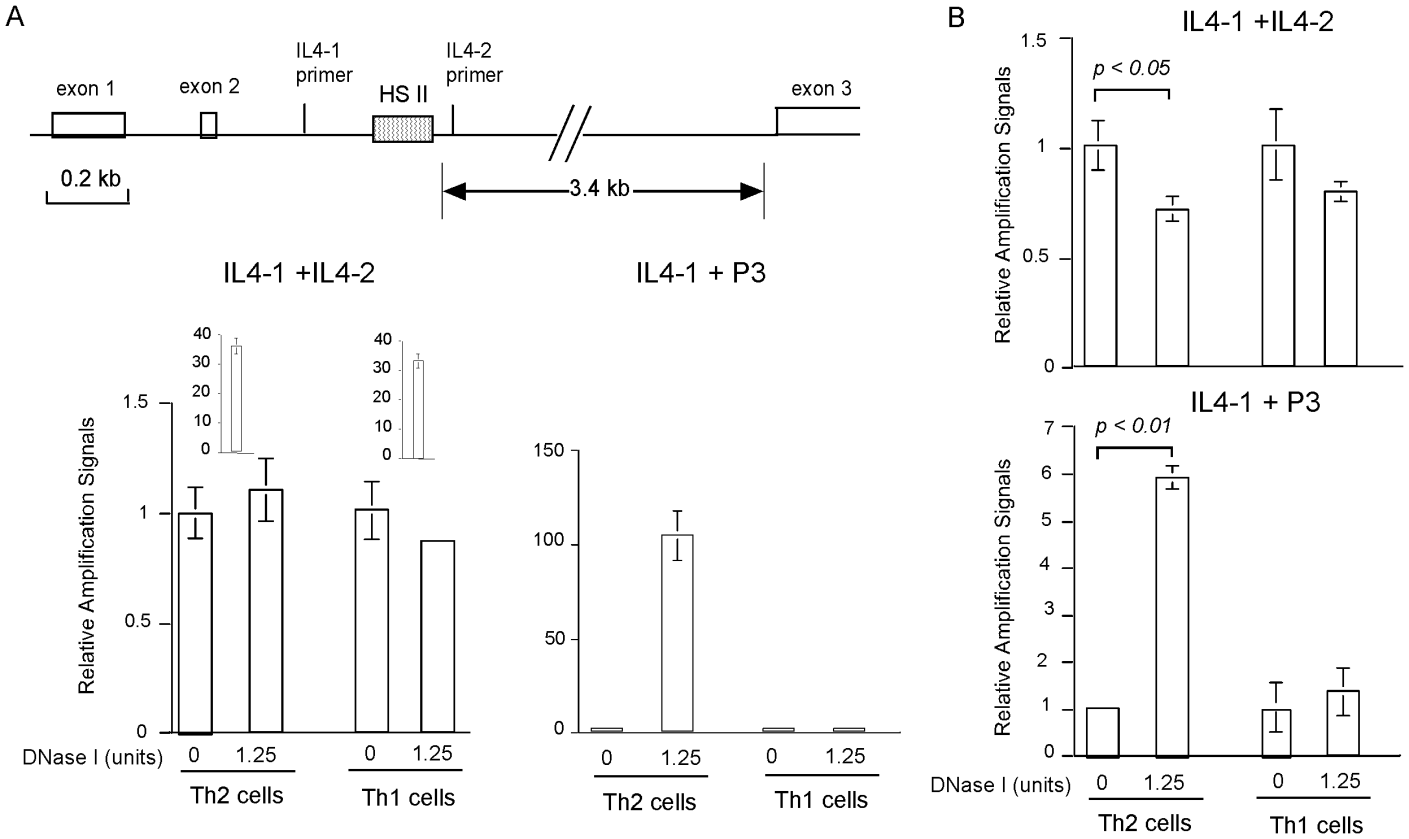
Detection of the HS II site of the *IL-4* gene in Th2 and Th1 cells. A. Detection of HS II site using DHS libraries as templates. Upper panel shows the positions of the HS II site and the PCR primers at the *IL-4* gene locus. The lower panel shows real-time PCR results using DHS libraries as templates and the indicated primer pairs. B. Detection of the HS II site using unpurified DNA as templates. Adaptor-ligated high-molecular-weight DNA derived from nuclei of Th2 and Th1 cells with or without DNase I digestion were used as templates in real-time PCR. Results with the indicated primer pair are shown. In all panels of the figure, relative amplification signals were determined by comparing to that of DNase I-undigested samples of the respective cell type.

To further validate the method in different cell type combination and DHS site, we performed similar experiments with Th2 and Th1 cells to detect a DHS site known as HS II at the *IL-4* gene, which is specifically present in Th2 but not Th1 cells [18]. The positions of the HS II site and the PCR primers derived from the *IL-4* gene locus are shown in Figure 8A upper panel. DHS libraries prepared from nuclei of Th2 and Th1 cells were used in real-time PCR analyses (Fig. 8A, lower panel). Like in the CD4 DHS experiment, the first PCR using primer pair (IL4-1 and IL4-2) encompassing the HS II site showed little amplification in all samples. In the second PCR with primer pair of IL4-1 and the adaptor-derived P3, robust amplification was detected in the DNase I digested Th2 sample but not others. Thus, the HS II site was specifically detected in Th2 but not Th1 cells.

We next tested whether the HS II site can be detected using DNA templates not purified by the magnetic beads. In this experiment, adaptor ligated high-molecular-weight DNA without subsequent sonication and beads separation were used directly as templates in real-time PCR. While no statistically significant difference between DNase I-digested and undigested Th1 samples was detected in the first PCR using primer pair of IL4-1 and IL4-2, a modest but statistically significant reduction of the amplification signal was observed in DNase I-digested compared to undigested Th2 samples (Fig. 8B, upper panel). However, the difference between DNase I-digested Th2 and Th1 samples was not statistically significant. The discrepancy underlines the unambiguity in detecting DHS site using unpurified DNA templates in combination with primers derived from endogenous genomic DNA sequences. In the second PCR using primer pair of IL4-1 and P3 (Fig. 8B, lower panel), strong amplification was detected in the DNase I-digested Th2 sample compared with the undigested Th2 sample although the fold of increase was not as large as that of the beads purified templates shown in Figure 8A. In contrast, no statistically significant increase of amplification signal in DNase I-digested over undigested Th1 samples. Therefore, the HS II site was detected by the second PCR specifically in Th2 cells.

## Discussion

This report describes a quick and reliable method of detecting DHS site in rare populations of cells. We developed the method using a previously characterized DHS site upstream of the murine *CD4* gene as a model system. We detected the presence of this DHS site in naïve CD4 Tcon cells and nTreg cells, a rare population of cells that comprises only 1–2% of total leukocytes in the secondary lymphoid tissues [17]. The method was further validated by experiments that detected a Th2 cell-specific DHS site HS II of the *IL-*4 gene. In this method, a biotinylated adaptor was attached to the genomic DNA ends created by DNase I digestion, and the DHS site was detected using two PCR reactions. In the first PCR, primers were derived from DNA sequences encompassing the DHS site. DHS site was detected by the absence or decrease of PCR products as the result of the cuts of the templates by DNase I between the primer sequences. In the second PCR, the presence of the DHS site was determined by positive PCR results using one primer derived from the sequence outside the DHS site and the other primer from the adaptor, which produced a smear of PCR products consisting of DNA of different sizes.

In DHS assays, there is usually a significant portion of genomic DNA that is left uncut by DNase I even at the DHS site. In previous PCR-based methods, the detection of a DHS site is based on quantitative difference in the amplification of the DNA sequence of the DHS site between DNase I-digested and undigested samples [12], [15]. In such assays, the large amount of DNA uncut by DNase I could mask the quantitative differences, leading to ambiguous results. The major improvement in the current method over the previous methods was that it overcame the interference by the genomic DNA undigested by DNase I at the DHS site. This was accomplished in two ways. First, DNA fragments with ends generated by DNase I digestion were isolated by magnetic beads. When such DNA was used as PCR template, the PCR results were largely free of the interference of DNase I-undigested DNA. Second, the second PCR employed a primer derived from the adaptor sequence. Since the sequence of this primer is not derived from the genome, PCR with such primer selectively amplifies only DNA with ends generated by DNase I and ligated to the adaptor while the uncut DNA is ignored. Our experiments showed that the combination of these two strategies ensured unambiguous detection of DHS sites. Moreover, even the second strategy alone appeared to be sufficient to detect a DHS site specifically, therefore may be regarded as a simplified version of the method.

Several other technical factors contributed to the reproducibility, specificity and sensitivity of the method. First, the nuclei were digested for 1 hr on ice instead of 10 min at 37°C as in most other protocols [19]. Such mild digestion conditions made it easier to control the digestion process, thus introduced less variations between experiments. Second, the genomic DNA was isolated and blunt-ended while embedded in gel, a technique used by Boyle et al. in genome-wide detection of DHS sites by deep sequencing [5]. This technique effectively minimizes random breaks of the DNA. After ligation of the adaptor, only high-molecular-weight DNA was recovered, then sonicated to an average size of 500 bp for magnetic beads isolation. The fragmentation of the genomic DNA decreased the “stickiness” of the genomic DNA, thereby reducing nonspecific binding of the DNA to the beads. In addition, we pre-treated the magnetic beads with yeast tRNA and BSA and incubated DNA and the beads in the presence of these two reagents to block nonspecific binding of DNA to the beads. More importantly, after the incubation, magnetic beads were treated with NaOH, which denatured the DNA bound to the beads and disrupted nonspecific binding of DNA to the beads. Collectively, these techniques increased the specificity of the method. Finally, to release the DNA from the low-melt agarose gel we digested the gel plug with ß-agarase, which in our experience greatly improved the yield of DNA. During ethanol precipitation, glycogen carrier was used to improve the yield as well. These techniques increased the sensitivity of the method. We have determined that as few as 0.2×10^5^ cells were sufficient for this method. Although we were not able to detect DHS site with fewer cells, it is reasonable to believe that the sensitivity could be further increased by incorporating radioisotope labelled nucleotide in the second PCR products and detecting the DNA smear by autoradiography.

## Conclusion

We described here a quick and reliable method for detecting DHS site in rare populations of cells. This method can be used to study DHS sites previously identified by genome-wide and traditional approaches in different cell types or under different experimental conditions. Under these circumstances, only certain pre-selected DHS sites need to be studied, making cost-prohibitive genome-wide analysis unnecessary. This method may also be modified for identifying novel DHS sites by designing PCR primers that walk a pre-determined region of genomic DNA. The method can be performed in any regular laboratory without the requirement for special equipment.
